# The SwAD-Task – An Innovative Paradigm for Measuring Costs of Switching Between Different Attentional Demands

**DOI:** 10.3389/fpsyg.2019.02178

**Published:** 2019-10-04

**Authors:** Magnus Liebherr, Stephanie Antons, Matthias Brand

**Affiliations:** Department of General Psychology: Cognition, University of Duisburg-Essen, Duisburg, Germany

**Keywords:** attentional demands, divided attention, selective attention, switching, paradigm

## Abstract

Task switching paradigms are frequently used to identify costs of switching between modalities, spatiality, attributes, rules, etc., but switching between different attentional demands has been somehow neglected. The present study introduces an innovative paradigm, that allows to test single attentional demands (such as selective and divided attention), and more importantly the process of switching between these demands. We examined the feasibility of the paradigm by focusing on the demands of selective and divided attention with a sample of 94 people (age: *M* = 21.44 years, *SD* = 2.68; 76 women). In addition, we tested correlations between the implemented single attentional demands and commonly used measures of selective and divided attention. Results show no general difference between individual assessments under single demand conditions. Reaction times under divided attention are significantly higher compared to selective attention. In the switching condition, reaction times in both demands increase with increased switching. Furthermore, switching costs significantly increase in selective but not in divided attention. Means of selective and divided attention in single and switching conditions significantly correlate with a commonly used measure of selective attention. Means of divided attention under single demand significantly correlate with performance in a commonly used dual-task paradigm. Summarizing the present findings, it can be stated that the introduced paradigm comprises a feasible way for quantifying the process of switching attention between different demands.

## Introduction

As our modern world is getting more and more complex, processes referred to as *cognitive control* become increasingly relevant. These processes comprise humans’ ability to quickly adapt to changes in the environment, to switch between different tasks, as well as to select appropriate actions, which are generally studied in task-switching paradigms (Monsell, 2003). Since its introduction (Jersild, 1927), task-switching paradigms are alternately described in terms of mental shifting, attention switching, attention shifting, or task shifting, but all refer to the same ability of performing a given task after having just performed a different one (for reviews see, e.g., Monsell, 2003; Kiesel et al., 2010; Koch et al., 2010). The resulting switching costs are commonly calculated by subtracting the average reaction times of non-switching from the average reaction times of switching (Aron, 2008). During recent years, the effects of (1) modality (e.g., Quinlan and Hill, 1999), (2) spatiality (e.g., Santangelo et al., 2010), (3) attributes (e.g., Longman et al., 2013), (4) rules (e.g., Chiu and Yantis, 2009), (5) stimulus sets or the relevant object (e.g., Gurd et al., 2002), and (6) response sets or applied operations to stimuli (e.g., Jäncke et al., 2000) on switching costs, have been studied extensively (for a review see Wager et al., 2004). However, switching between different attentional demands, such as selective attention, divided attention, vigilance, etc. – which is common in daily life – is somehow neglected. For example, when thinking about an everyday morning, it becomes apparent that brushing teeth while checking mails, having breakfast while reading the newspaper and helping the children get dressed while calling a colleague are all situations that require the ability to divide attention. In the next moment, sitting in the car and driving on the highway to work for more than an hour requires to maintain attention over prolonged periods of time with only a low degree of attention (vigilance). Once having arrived at the office and sitting at the desk concentrating on a specific task while ignoring everything else can be referred to as selective attention. The study at hand aimed to investigate the ability of switching between the most relevant attentional components of our everyday life, selective and divided attention, based on the general idea of task-switching paradigms.

Converging evidence from previous task-switching studies report increased response times and error rates in switching compared to repeating conditions (Kiesel et al., 2010; Vandierendonck et al., 2010). Furthermore, studies on the bivalent effect show increased costs in switching between bivalent stimuli compared to switching between univalent stimuli (e.g., Rubin and Meiran, 2005). Bivalent stimuli are characterized by different meanings, such as colored even and odd numbers, whereas univalent stimuli constitute stimuli with a single meaning, such as colored bars (e.g., Metzak et al., 2013). During recent years, numerous studies showed that responses on univalent stimuli still decrease when bivalent stimuli appear occasionally among them (Meier et al., 2009, 2013; Grundy et al., 2011). Woodward and colleagues argue that this decline is based on a more cautious response style because of the perceived “trickness” (Woodward et al., 2003, 2008). Furthermore, Kray and Lindenberger (2000) suggest that bivalent stimuli permit different actions, causing to uncertainty, and therefore increase the time for making a response.

More generally, theoretical assumptions on task-switching suggest that switching costs reflect both endogenously and exogenously driven cognitive control process. Rogers and Monsell (1995) assume that the cognitive system needs a certain time to remove task set parameters relevant from the previous trial and replace them with parameters relevant for the current trial, which leads to a time-consuming process of *task set reconfiguration*. However, the authors identified decreased switching costs with increased preparation time prior to the target onset. More recent studies consistently confirmed the finding of decreased switching costs with an increased time to prepare for a forthcoming switch (e.g., Meiran, 1996; Hunt and Kingstone, 2004; Gade and Koch, 2007; Wylie et al., 2009; Kiesel et al., 2010). Furthermore, studies indicated a substantial asymptote in switching costs after approximately 600 ms of preparation, but substantial costs have also been reported even after 5 s of preparation or more (e.g., Kimberg et al., 2000; Sohn et al., 2000). The residual switching costs, which reflect a fundamental limit of reconfiguration, are explained by the influence of task stimuli (e.g., Rubinstein et al., 2001; Mayr and Kliegl, 2003). Next to task set reconfiguration, Allport and colleagues (Allport et al., 1994; Allport and Wylie, 2000) stated the task *set inertia hypothesis*. In contrast to Rogers and Monsell (1995), the authors explain switching costs based on familiar memory processes, such as interference and priming (Altmann, 2002, 2003; Altmann and Gray, 2002, 2008). Here, the authors assume that switching costs reflect the time taken to establish the desired task-set and to resolve interferences resulting from persisting activation of the previous task and the negative priming of relevant task (e.g., inhibition). According to this hypothesis, switching costs indicate the extent of interferences from the previous but not the subsequent task. Furthermore, this account explains the remaining residual switching costs by proactive interference from elements of the previous task instead of the preparation time (see Vandierendonck et al., 2010). In addition to task set reconfiguration and the task-set inertia hypothesis, Meiran et al. (2000, p. 211) suggest “that switching costs consist of three components reflecting (1) the passive dissipation of the previous task set, (2) the preparation of the new task set, and (3) a residual component”.

Based on previous findings, we developed an innovative paradigm – the **Sw**itching **A**ttentional **D**emands-task (SwAD-task) – which allows to investigate the process of switching between different attentional demands (e.g., selective attention, divided attention, vigilance, and sustained attention). Within the present version of the paradigm, the focus is on the consideration of selective and divided attention. In contrast to bivalent stimuli, which are characterized by different meanings but only one defined as a target, tasks of divided attention involve multi-expressions of one stimulus or multiple stimuli at one time that need to be responded to (multiple response options). Therefore, the color of the numbers in the context of bivalent stimuli, where participants have to respond – example – to even numbers, acts as a distractor, whereas in conditions of divided attention, participants need to react to a specific object as well as a specific number, presented simultaneously (for e.g., by pressing to different buttons). However, based on findings on the bivalent effect but also studies on task-switching, we hypothesize that increased switching between selective and divided attention leads to a deteriorated performance in both tasks. Furthermore, we expect a decreased performance in switching conditions compared to single demand conditions, as shown in previous studies of modality, spatiality and others. We additionally test correlations between the involved attentional tasks and commonly used methods for quantifying selective and divided attention.

## Materials and Methods

### Participants

In total, 94 people (age: *M* = 21.44 years, *SD* = 2.68; 76 women) completed the SwAD task. A subsample of 74 participants (age: *M* = 21.65 years, *SD* = 2.72; 57 women) further completed the two tasks for selective and divided attention. All participants reported normal or corrected-to-normal eyesight and hearing as well as no history of neurological or psychological disorders. Participants were recruited at the University Duisburg-Essen. The study was performed in accordance with the ethical standards laid down in the Declaration of Helsinki and approved by the local ethics committee of the Department of Computer Science and Applied Cognitive Sciences, University Duisburg-Essen. Prior to the experiment, all participants provided written informed consent and were informed that they could end participation at any time without reprisal.

### Materials

The *SwAD-task* offers the possibility to investigate different attentional demands, but more importantly, the ability of switching in between them. The modular character of the task – which is based on the Stimulus Delivery and Experiment Control Software, Presentation^®^ – allows the user to address different attentional demands. In the version at hand, we implemented the attentional demands of selective and divided attention. Typically, the SwAD-task comprises three conditions: (1) Training, (2) Single demand, and (3) Switching demand (see Table 1).

**Table 1:** Different conditions in the SwAD-task.

(1) Training	(2) Single demand	(3) Switching demand
1. Two blocks 2. 10 trials/block 3. Feedback	1. Four blocks of each attentional demand 2. Twenty-six trials/block 3. Five to eight target stimuli/block 4. Two min resting period between different attentional demands	1. Four blocks of each attentional demand performed alternately 2. Twenty-six trials/block 3. Five to eight target stimuli/block 4. No resting period between the blocks

**Figure 1:**
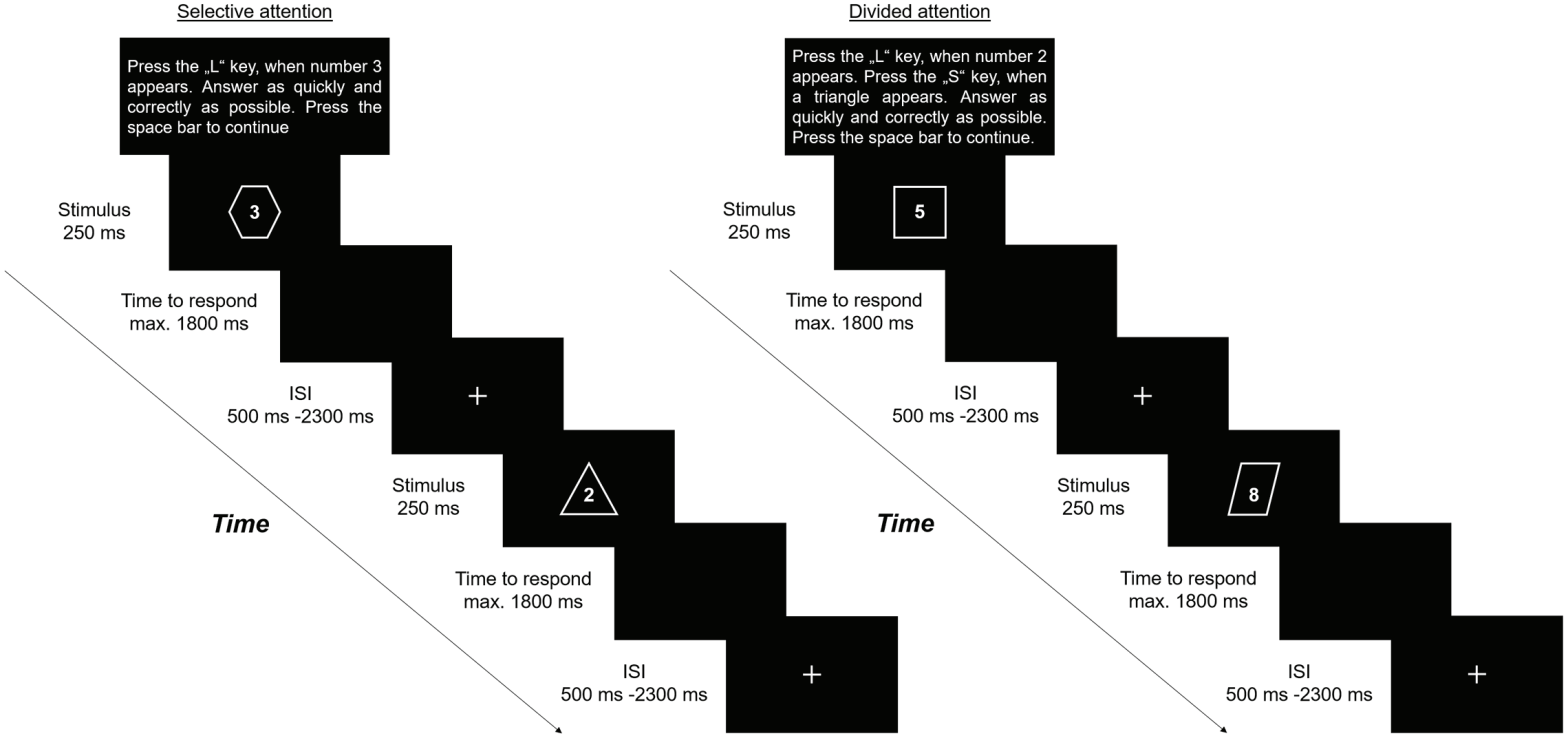
Schematic overview of the SwAD-task – sequences in either selective or divided attention, depending on the instructions.

One stimulus consists of a figure (e.g., triangle) and a number (e.g., 3). The distinction between selective and divided attention tasks results from the instructions, presented prior to each block. In selective attention, participants are asked to respond to one pre-defined target which was either a number or a figure by pressing one button (e.g., Key: L) while ignoring the other part of the stimulus. By contrast, in divided attention, a number and a shape were defined as targets. Here, participants should respond by pressing one button for target numbers (e.g., Key: L) and one button for target figures (e.g., Key: S). In the training session, participants perform two blocks – one block of selective and one of divided attention – each comprising 10 trials. Feedback on whether participants respond right, incorrect to a target stimulus, incorrect to a non-target stimulus, or with the wrong button, is presented only in the training condition. Single demand conditions comprise four blocks of selective and four blocks of divided attention with 2 min resting between the respective conditions. In the switching condition, four blocks of each attentional demand need to be performed alternately. Blocks in single and switching conditions consist each of a total number of 26 trials, including five to eight randomized target stimuli. To avoid spatial biases, numbers (1–9), and shapes (triangle, rhombus, rectangle, circle, star, and octagon) are presented simultaneously in the middle of the screen in white font against a black background. We used Apercu Mono font (50 pt.), in which all letters and numbers have the same width to illustrate the numbers. All shapes are presented with similar size (height: 600–750px; width 600–700px). The maximum time to respond is set to 1,800 ms. Target stimuli as well as response buttons in the divided attention condition change randomly from block to block. Each stimulus is presented for 250 ms. The interstimulus interval is randomized between 500 and 2,300 ms and starts after participants respond or 1,800 ms in case of no response. During the interstimulus interval, a fixation-cross is presented in the middle of the screen (see also Figure 1). To avoid modality effects, the stimuli are only presented visually and response is limited to manually. Task performance is quantified by measuring reaction times. Time to complete the SwAD-task took about 20 min.

The correlation for selective attention was tested with an auditory *oddball task* (e.g., Squires et al., 1975; Fichtenholtz et al., 2004; Huettel and McCarthy, 2004). The version at hand comprises a short training trial (six stimuli, two target/four non-target), as well as a testing trial with 20 target tones (piano) and 90 non-target tones (horn), presented in a random order. Each stimulus has the same length (200 ms) and volume. Participants have to respond to target tones by pressing the space bar within a time window of 2,000 ms. The interstimulus interval is randomized between 500 and 1,000 ms. Task-performance is quantified by reaction times. To test correlations for divided attention, we used a c*lassic dual-task paradigm*. The paradigm consists of an auditory choice reaction task and a simple visual reaction task (modified version of Wegmann et al., 2017). Within the auditory choice reaction task, four different tones, one defined as target and one as non-target are presented. Tones are randomized across four blocks. Within each block, participants have to respond to five target tones by pressing the arrow down button while ignoring the remaining five. Two target-stimuli do not follow each other. Target and non-target stimuli change randomly across the blocks. Inter-stimulus intervals are randomized between 1,750 and 4,000 ms. Each tone has the same length (1,150 ms) and volume. The visual task is performed in parallel. Within each block, four to six black dots (diameter: 300 px) are presented between the tones. Visual stimuli are presented in a time span of 1,750 to 11,250 ms. Participants have to respond by pressing the arrow down button whenever they see a black dot. Time to respond is limited to 2,500 ms in the auditory task and 1,750 ms in the visual task. Error rates are measured for the auditory and visual task.

### Procedure

After carefully reading the instructions, participants gave informed consent and started with the SwAD-task. All participants were examined individually. To test for potential sequence effects, we randomized the order of the conditions, except for the training run, which was always set at the beginning. The following six sequences were used with equal distribution: selective/divided/switching, selective/switching/divided, divided/selective/switching, divided/switching/selective, switching/selective/divided, and switching/divided/selective.

Between the SwAD-task and the oddball task, participants had a 5-min break to rest. Afterwards, participants performed the classic dual-task paradigm. The tasks were performed by all participants in the same order. All visual tasks were presented on a conventional screen with a size of 24 inches. For the auditory tasks, we used two commercial loudspeakers, both standing in front of the participant. The volume, the position of the chair, the screen, and the loudspeakers were standardized and cross-marked to ensure equal conditions for all participants.

### Data Analysis/Statistical Analysis

To identify potential costs due to switching between different attentional demands, mean reaction times for selective and divided attention in single demand and switching demand conditions were calculated. Prior to mean calculations, reaction times of each block that exceeded two standard deviations of participants’ mean of the respective block were identified as outliers. This concerned less than two reaction times per condition and participant. Only correct responses were included in mean calculations. None participant had more than 50% errors (miss and false answer) in any block. Mean reaction times for each block were calculated. We analyzed reaction times of each bock for the single demand conditions and the switching demand conditions, using analyses of variance with repeated measures (ANOVA), with “type of attentional demand” (selective/divided) and “block” (block 1–4) as within-subject factors. In the next step, mean reaction times were calculated for each condition. In the single demand conditions, mean reaction times were calculated over all four blocks. In the switching demands condition only block 2–4 were included in the mean calculation, since the first blocks of each demand does not include effects of switching. Furthermore, we used ANOVAs with repeated measures to analyze reaction times in single and switching demands conditions with the within-subject factors “type of attentional demand” (selective/divided) and “condition” (single demand/switching demand). Switching costs for selective and divided attention were calculated via the difference between the switching demand and single demand conditions. Furthermore, we used Pearson correlations to test correlations between single demand conditions and commonly used measures. Differences between correlations were evaluated using Fisher’s *Z*.

## Results

### Blocks of Single Demand and Switching Demand Conditions

**Figure 2:**
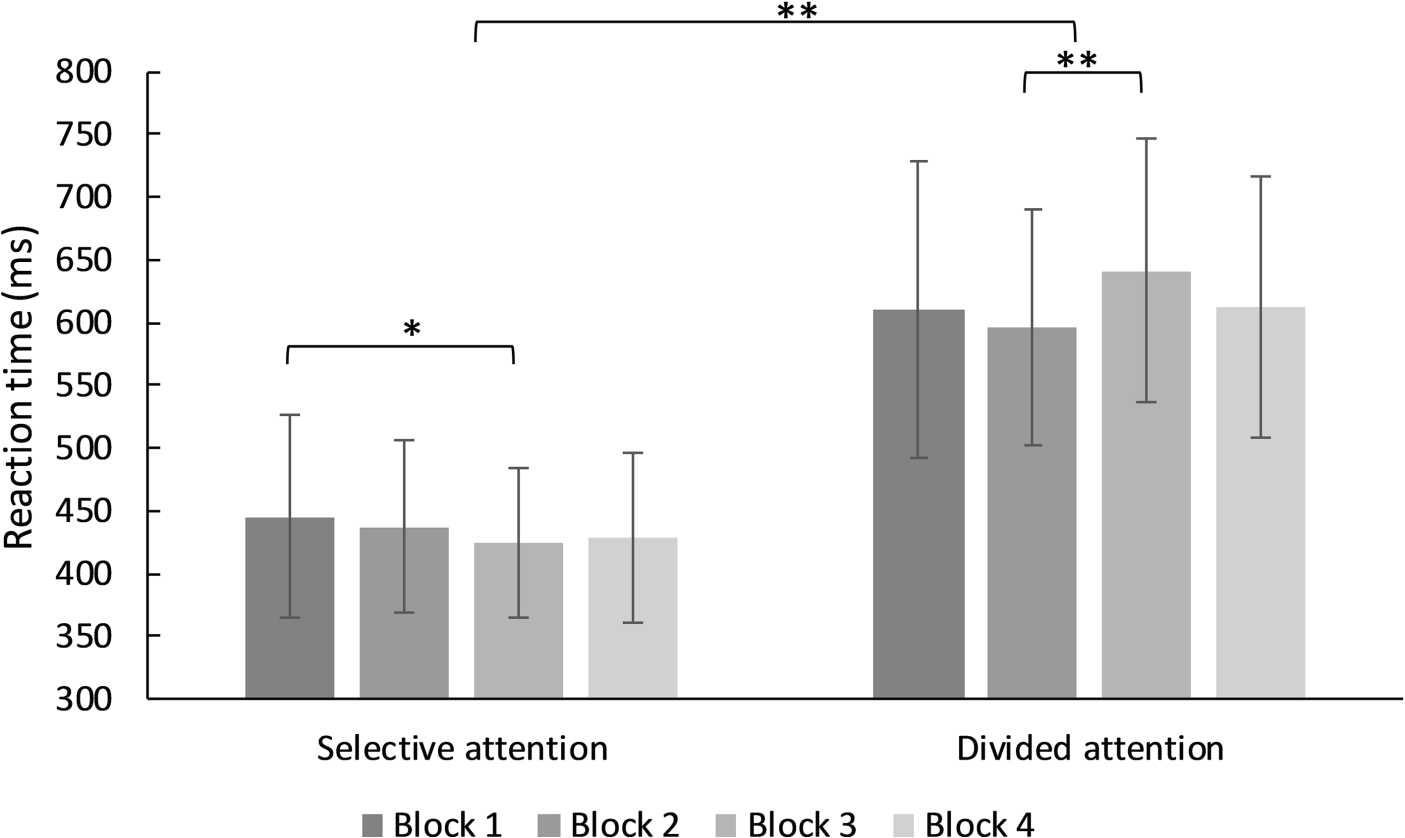
Mean reaction times for trials in the selective and divided single demand condition. ^*^p < 0.05, ^**^p < 0.001.

There was a significant main effect for type of attentional demand (selective, divided), *F*(3, 93) = 675.87, MSE = 9,176.31, *p* < 0.001, partial *η*^2^ = 0.879. Reaction times in the divided attention condition were higher than in the selective attention condition. There was no significant main effect for blocks *F*(3, 93) = 2.31, MSE = 4,114.52, *p* = 0.077, partial *η*^2^ = 0.024, but a significant interaction effect between the factors type of attentional demand and block, *F*(2.73, 253.66) = 6.79, MSE = 5,076.68, *p* < 0.001, partial *η*^2^ = 0.068. In the condition of selective attention, reaction times differed significantly but with low effect size between the first and third block, *M*_dif_ = −21.08, *t*(93) = 2.71, *p* = 0.008, *d* = −0.25, CI [−0.54, 0.04]. In the divided attention condition, reaction times of the second and third block differed significantly, *M*_dif_ = −44.36, *t*(93) = −4.32, *p* < 0.001, *d* = 0.47, CI [0.18, 0.76] (see Figure 2, for further details).

**Figure 3:**
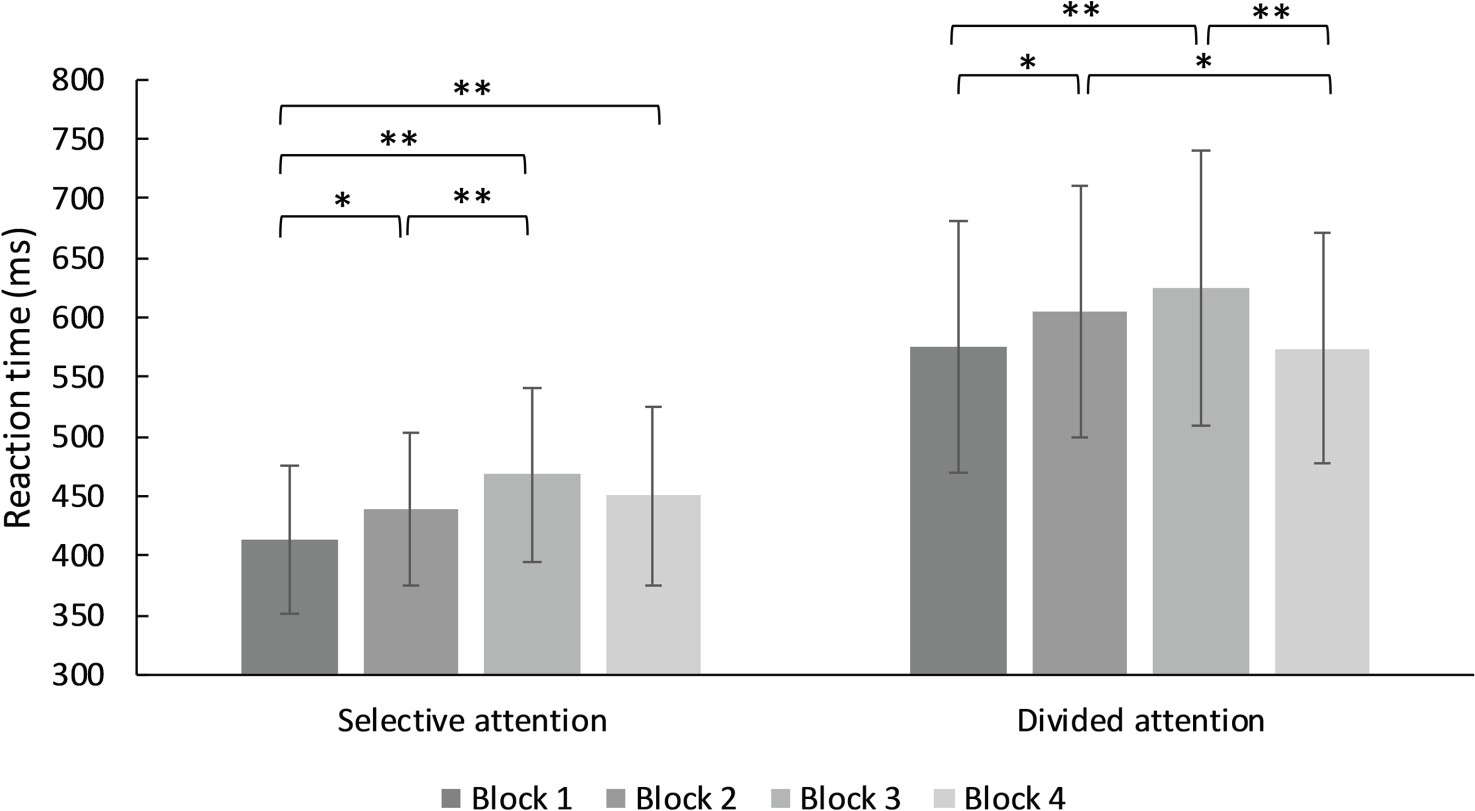
Mean reaction times for trials in the selective and divided switching demands condition. ^*^p < 0.05, ^**^p < 0.001.

In the switching demand condition, there was a significant main effect for type of attentional demand (selective vs. divided), *F*(1, 93) = 803.72, MSE = 5,441.00, *p* < 0.001, *η*^2^ = 0.896. Reaction times in divided attention demand were significantly slower. There was a main effect of block, *F*(3, 93) = 20.19, MSE = 4,330.66, *p* < 0.001, *η*^2^ = 0.178. Reaction times increased from block 1 to 3 except from 3 to 4 in selective and divided attention. There was a significant interaction effect between demand and block, *F*(3, 91) = 4.37, MSE = 4,046.95, *p* = 0.005, *η*^2^ = 0.045 (see Figure 3, for further details).

### Switching Costs

**Figure 4:**
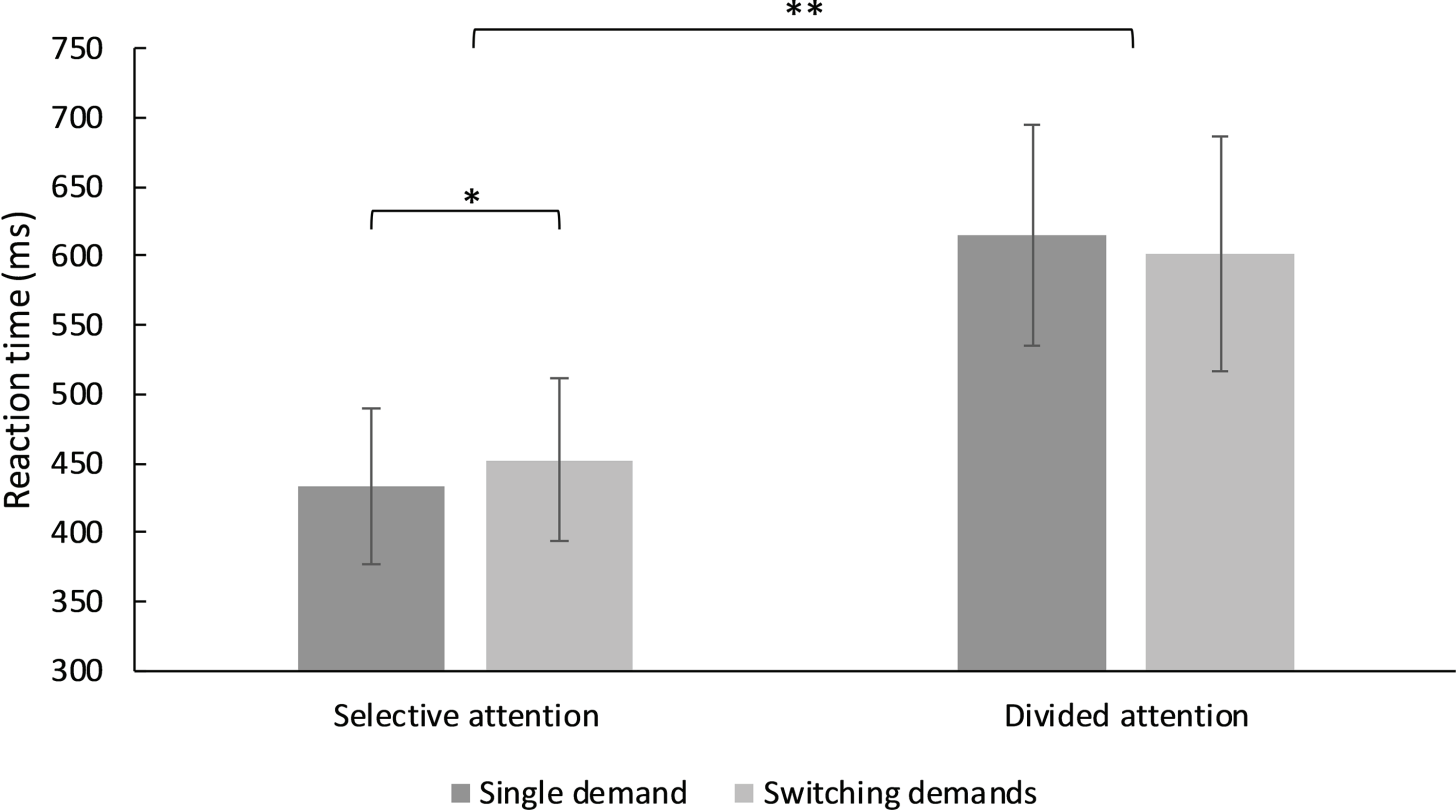
Mean reaction times for selective and divided attention in the single demand and switching demands condition. ^*^p < 0.05, ^**^p < 0.001.

There was no main effect of switching, *F*(1, 93) = 0.44, MSE = 1,846.43, *p* = 0.511, *η*^2^ = 0.005, but of type of attentional demand, *F*(1, 93) = 1,120.93, MSE = 2,295.56, *p* < 0.001, *η*^2^ = 0.923. There was also a significant interaction between type of attentional demand and switching, *F*(1, 93) = 15.80, MSE = 24,603.50, *p* < 0.001, *η*^2^ = 0.145. Figure 4 shows the interaction effects: Reaction times for selective attention were significantly slower in the switching demands condition compared to the single demand condition, *M*_dif_ = −19.11, *t*(93) = −3.85, *p* < 0.001, *d* = 0.42, CI [0.12, 0.70]. By contrast, reaction times for divided attention were slightly faster in the switching demand condition compared to the single demand condition, however, this effect did not reach significance, *M*_dif_ = 13.25, *t*(93) = 1.92, *p* = 0.058, *d* = −0.20, CI [−0.49, 0.08]. The direct comparison between switching costs revealed a significant difference, *M*_dif_ = 32.36, *t*(93) = 3.97, *p* < 0.001, *d* = −0.61, CI [−0.90, −0.32]. Since the order of conditions was randomized over participants we also included the randomization as covariate into the model which did not affect the results, *F*s(1, 93) < 1.31, *p*’s > 0.255.

### Correlations With Common Measures of Selective and Divided Attention

Descriptive statistics and correlations between SwAD mean reaction times and oddball as well as dual task performances for the subsample are presented in Table 2. There are high correlations between selective and divided attention (in single demand and switching demand conditions) and oddball task performances. In the switching condition, comparisons between correlations revealed a higher correlation between the oddball task performance and types of attentional demands for selective attention compared to divided attention, Fisher’s *Z* = 2.32, *p =* 0.010. The dual task performance solely correlated with divided attention in the single demand condition. The correlations between the dual task performance and types of attentional demands in the switching condition were also higher for selective attention compared to divided attention, Fisher’s *Z* = 2.04, *p =* 0.021.

**Table 2:** Correlations with common measures of selective and divided attention.

	*M*	*SD*	(1)	(2)	(3)	(4)	(5)
1. Single demand: selective attention	437.68	55.25	–				
2. Single demand: divided attention	613.70	81.24	0.533^**^	–			
3. Switching demands: selective attention (trial 2–4)	452.22	57.67	0.593^**^	0.654^**^	–		
4. Switching demands: divided attention (trial 2–4)	596.41	70.83	0.573^**^	0.671^**^	0.754^**^	–	
5. Oddball	597.62	93.24	0.477^**^	0.476^**^	0.532^**^	0.363^**^	–
6. Dual-task	9.03	6.32	0.118	0.276^*^	0.185	0.180	0.054

*N = 74*.

* *p < 0.05*

** *p < 0.001*.

## Discussion

In the present study, we introduced a new paradigm for switching attention. While previous paradigms focused on effects of modality, spatiality, attributes, rules, etc., the SwAD-task addresses switching between different attentional demands. We tested the paradigm in a sample of healthy young adults and compared the findings against results of commonly used paradigms in this field. Therefore, the discussion comprises two major parts, one focusing on the findings of the task itself, and the other discussing correlations with commonly used tasks of selective and divided attention. Considering the SwAD-task itself, the following major findings can be summarized:

1. In single demand conditions, there are no general differences between individual blocks, but reaction times under divided attention are significantly higher compared to selective attention.
2. In the condition of switching attentional demands, reaction times increased with increased switching, except in the last block.
3. Switching costs significantly increased in selective, but not in divided attention. However, switching generally seems to have a beneficial effect on divided attention.

Considering the longer reaction times in divided vs. selective attention, one might argue that it is obvious that performance decreases as soon as an additional demand is added. However, it is not as simple as it seems, as we demonstrated in a previous EEG study that additional simple motor demands can have beneficial effects on performing a primary cognitive task (Liebherr et al., 2018). In accordance with previous studies, we assume a few aspects relevant for the different reaction times between selective and divided attention. One aspect might be the exclusive use of visual stimuli. We suggest that focusing on two kinds of visual stimuli – as in divided attention – exceeded the perceptual resources available. Early evidence for this explanation comes from Sutton et al. (1965) and Wickens et al. (1983), who pointed out the relevance of the extent to which two tasks/stimuli compete for a common resource. More recently, Talsma et al. (2006) demonstrated decreased attentional capacity for processing stimuli in the same modality rather than different modalities. By contrast, Spence and Driver (1997) as well as Spence et al. (2001) hold the view that perception of stimuli increases when attending to the same modality, relative to different modality. Further potential explanations for differences in reaction times between selective and divided attention come from imaging studies. For example, Weerda et al. (2006) reported higher activity in lateral prefrontal and posterior parietal cortex under divided attention than during selective attention, both areas that are also associated with top-down control (e.g., Buschman and Miller, 2007). The assumption of increased top-down control in divided attention contributes to increased reaction times. We further assume that the attentional spotlight provides an additional plausible explanation for these differences (Posner et al., 1980). While early spotlight theories argue that humans can direct attention to only one location at a time (Posner et al., 1980; Eriksen and James, 1986), McMains and Somers (2004) provided neural evidence for the ability to split attention between two different locations. However, there is still a lack of evidence regarding the costs for splitting spatial attention but also regarding the amount of spatial locations that humans are able to simultaneously attend to. Studies which indicate a rapid redeployment of a serial spotlight of attention reported a time cost of 200–500 ms (e.g., Reeves and Sperling, 1986; Weichselgartner and Sperling, 1987; Duncan et al., 1994; Moore et al., 1996; Peterson and Juola, 2000). In addition to the three relevant aspects of (1) competition for a common resource, (2) top-down processes, and (3) a serial spotlight, which argue from a perception perspective, the response stage should be considered as a fourth aspect. Here, it can be assumed that the different numbers of response buttons (one in the selective attention task and two in the divided attention task) might also be responsible for the differences in response times. Considering this processing phase, which is more closely connected to motor actions, we argue that the demand for attention increases as more appropriate actions are needed.

The fact that individual blocks in respective conditions do not differ from each other – except block 2–3 in divided attention and trial 1–3 in selective attention – suggests that neither learning nor fatigue effects occurred. Especially in traditional attention research, there is a risk that people get bored or fatigued during the testing because of relatively simple stimuli and long duration times. By contrast, Ackerman and Kanfer (2009) suggest that – in a healthy young sample – the aspect of fatigue during long cognitive testing can be neglected. Based on their findings, the authors reported greater effects of personality, interest, motivation, and trait complexes then test-length. Nevertheless, this aspect should not be neglected in samples comprising older people or people with neurodegenerative diseases, as it has been shown in previous studies (e.g., Schwid et al., 2003).

Regarding the core aim of the present study of testing the effects of switching between attentional demands, we argue that switching adversely affects selective attention. One mechanism that may provide further understanding is the ability of inhibition. Especially in tasks where a certain stimulus needs to be focused and distractors have to be ignored, mechanisms of inhibition play a crucial role. For example, Booth et al. (2003) point out that a decreased performance in tasks of selective attention results either from a deficit in sustained attention or a failure to inhibit a pre-potent response. Furthermore, Monsell (2003) describe increased switching costs by carry-over effects of task-set activation and inhibition. Based on previous findings of inhibition, two possible approaches should be discussed in the present context. First, we assume that the reduced need for inhibition in divided attention leads to a decreased inhibition control in tasks of selective attention, in terms of carry-over effects, such as proposed by Monsell (2003). Along with Goschke (2000), who suggest that the amount of response conflict adjusts the degree of inhibition, it can be assumed that more response options – as it was the case under divided attention – lead to a lower level of required inhibition. Second, various studies – that investigated the role of inhibition in task switching – propose proactive interferences resulting from continued priming of the previous task and suppression of the currently intended task, responsible for switching costs (e.g., Allport et al., 1994; Allport and Wylie, 1999; Yeung et al., 2006). One might assume that switching between tasks of selective and divided attention would lead to similar switching costs in both demands, which contrasts with the present findings. Therefore, a third approach concerning “switching-cost asymmetries,” needs to be additionally considered. This phenomenon proposes that switching to the “weaker task” leads to less switching costs (e.g., Allport et al., 1994; Allport and Wylie, 1999, 2000). For example, within their fifth experiment, Allport et al. (1994) report higher switching costs for word naming (strong task) compared to the “weaker” color naming task. In the studies by Allport and colleagues, it is relatively clear to identify the strong and the weak task, whereas in the present study it is somehow more difficult. Along with Koch et al. (2010), who summarize that switching-cost asymmetries do not necessarily imply inhibition, it can be assumed that other mechanisms additionally characterize the difficulty of a task. For example, effects of practice (e.g., McDowd, 1986) as well as the use of new technologies (e.g., Strobach et al., 2012) on divided attention, suggest that younger adults – as tested in the study at hand – have fewer problems with dividing their attention compared to selectively focusing it. By contrast, one might argue that divided attention requires increasing working-memory resources, which leads to an increased difficulty (cf., Barch et al., 1997). In this case, the present findings would be in contrast to the phenomenon of “switching-cost asymmetries,” proposed by Allport and colleagues (Allport et al., 1994; Allport and Wylie, 1999, 2000). However, Yeung and Monsell (2003) also report larger costs of switching to the weaker task but Monsell et al. (2000) conclud that it is not possible to generalize this phenomenon. Wrapping up, various aspects that may explain the current findings are theoretically plausible. It can be summarized that processes of inhibition, as well as carry-over effects and differences in task difficulty might be highly relevant in this context. Considering neural activation patterns of selective and divided attention as well as attentional switching might provide a deeper understanding of the findings at hand. For example, Nebel et al. (2005) report a widespread network including dorso- and ventrolateral prefrontal structures, superior and inferior parietal cortex, and anterior cingulate gyrus, activated under divided and selective attention. Under divided attention, activity was enhanced, and left-sided homologues were recruited. Such alike was identified under conditions of selective attention as soon as the authors increased the complexity of the task. Findings by Hahn et al. (2008) revealed no specific functional brain activity for divided attention. By contrast, Johnson and Zatorre (2006) suggest two distinct neural processes. The authors report a primary modulation of sensory cortices for achieving selective attention, whereas divided attention recruited structures of the middle-dorsolateral prefrontal cortex. Weerda et al. (2006) report higher activity in lateral prefrontal and posterior parietal cortex under divided attention than during selective attention. Within recent years, structures of the middle frontal gyrus have frequently been attributed to processes of divided attention (Herath et al., 2001; Corbetta and Shulman, 2002; Schubert and Szameitat, 2003; Johnson and Zatorre, 2006; Stelzel et al., 2006; Johnson et al., 2007; Moisala et al., 2015; Salo et al., 2015, 2017). For example, Herath et al. (2001) identified activity in the right inferior frontal gyrus as soon as the inter-stimulus interval was <300 ms. While one might have suspected that divided and switching attention underlie similar activation pattern – responsible for the differences in switching costs between selective and divided attention – Herath et al. (2001) provide clear evidence for two distinct neural processes. Furthermore, direct evidence comes from a meta-analysis conducted by Wager et al. (2004). The authors report structures of the medial prefrontal, superior and inferior parietal, medial parietal, and premotor cortices attributed to processes of switching attention. Therefore, it can be summarized that a common mechanism might be relevant for selective and divided attention but switching attention attributes to distinct neural mechanism.

However, we found different results in switching costs of selective vs. divided attention; both demands indicated increased reaction times with increased switching, except the last blocks. Therefore, it can be assumed that with increased switching, carry-over effects become increasingly relevant in both demands. As discussed above, the extent to which different mechanisms such as inhibition or task-set activation are involved in each condition leads to carry-over effects. For example, Monsell (2003) highlight the carry-over effect as an important contributor to switching costs but stated that it is unclear whether the slowing of task-specific processes or the trigger of extra control processes leads to the effect. Further evidence suggests a carry-over of priming effects from previous tasks, responsible for a prolongation of reaction times (Hsieh and Yu, 2003; Hsieh and Liu, 2005). The improvements in reaction times in the last blocks of selective and divided attention (in switching condition) may indicate that people got familiar with the switching or training effects occurred. Therefore, it could be speculated that further switching would result in further improvements. However, switching between the very same tasks does not occur in our daily lives. Therefore, in future studies, the paradigm should be tested with more complex stimuli, which should be changed randomly, but switching between attentional demands should remain the same.

Regarding the stated procedure of the discussion, we would like to briefly discuss the results of the SwAD-task against common measures of selective and divided attention: it can be summarized that:

4. Means of selective and divided attention in both single and switching conditions significantly correlate with a commonly used measure of selective attention.
5. Means of divided attention under single demand significantly correlate with performance in a commonly used dual-task paradigm.

Findings from the oddball task – which was previously used in an uncountable number of studies – in relation to the results from the SwAD-task, provide indirect evidence of a common mechanism or an overlapping between selective and divided attention. This assumption is in accordance with previous imaging studies – already stated above – that indicated common neural structures activated under selective and divided attention (e.g., Nebel et al., 2005; Weerda et al., 2006). The significant correlation of divided attention with findings from the dual-task paradigm exclusively in the single demand condition, once again suggests the existence of carry-over effects. As already discussed – at different places of the manuscript – it can be suggested that switching between different demands leads to a transfer of mechanisms relevant in one but not in the other demand (Hsieh and Yu, 2003; Monsell, 2003; Hsieh and Liu, 2005). Furthermore, the used dual-task paradigm consisted of an auditory and visual task, whereas the divided attention task in the SwAD-task comprised only visual stimuli. In addition, in the dual-task paradigm, we used a simple reaction task as visual task. Therefore, we assume increased requirements of working memory capacity in divided attention task of the SwAD-task compared to the dual-task paradigm, which is additionally affected by the switching (see also Baddeley et al., 2001; Lépine et al., 2005). Future studies should investigate the effects of executive functions, working memory but also individual attributes such as age. However, the study at hand – to our knowledge – is the first to investigate the specific effects of switching attentional demands. Based on findings from previous switching studies, we tried to eliminate possible biases, such as modality and spatiality effects. Furthermore, we tested sequence effects by presenting the three conditions of single demand – selective attention, single demand – divided attention, and switching demands in a randomized order. Results suggest that there is no effect on the order in which individual conditions are presented. In contrast to previous switching paradigms, we used relatively long times for switching between different demands. As we stated in the introduction, increased time to prepare leads to decreased switching costs, albeit switching costs were reported even after approximately 600 ms of preparation time (Rogers and Monsell, 1995; Meiran, 1996; Kimberg et al., 2000; Sohn et al., 2000; Hunt and Kingstone, 2004; Gade and Koch, 2007; Wylie et al., 2009). Therefore, future studies in the context of switching between attentional demands should consider this aspect by varying the time for preparation. In order to address the decreased reaction times in the last blocks of the switching condition, effects of an increased number of blocks but decreased length should also be investigated. Since the study at hand first considered the aspect of switching between attentional demands, we quantified switching costs based on reaction times as well as on a block level, which constitutes the comparison between switching blocks and single-demand blocks (e.g., Kray and Lindenberger, 2000; Kray et al., 2002; Wasylyshyn et al., 2011). By contrast, switching between trials focuses on the comparison of switching trials and repeated trials (e.g., Rogers and Monsell, 1995; Meiran, 1996; Kray et al., 2002). Therefore, future studies should additionally focus on the error rates as well as switching costs on a trial level. Next to its limitations, the present study provides an innovative paradigm, contributing to a better understanding of switching attention. Behavioral findings of the present study – that provide evidence for the feasibility – should be supplemented with neurophysiological measures, such as EEG or fMRI, to get a better understanding of the underlying mechanisms. Furthermore, future studies should test modified versions of the paradigm, focusing on switching between further attentional demands, such as vigilance or sustained attention.

## Data Availability Statement

The datasets generated for this study are available on request to the corresponding author.

## Ethics Statement

The studies involving human participants were reviewed and approved by Ethics Committee of the Department of Computer Science and Applied Cognitive Sciences. The patients/participants provided their written informed consent to participate in this study.

## Author Contributions

All authors contributed equally to the manuscript. However, ML had the idea, SA was responsible for the analysis, and MB coordinated the study.

### Conflict of Interest

The authors declare that the research was conducted in the absence of any commercial or financial relationships that could be construed as a potential conflict of interest.
